# The impact of uncorrected myopia on individuals and society

**Published:** 2019-05-13

**Authors:** Nathan Congdon, Anthea Burnett, Kevin Frick

**Affiliations:** 1 Ulverscroft Chair of Global Eye Health: Centre for Public Health, Queen's University Belfast & Orbis International & Sun Yat-sen University, Guangzhou, China, Royal Victoria Hospital, Belfast, Ireland, UK.; 2 Global Research Manager: Brien Holden Vision Institute, Sydney, Australia.; 3 Professor and Vice Dean for Education: Johns Hopkins University, Carey Business School, Baltimore, USA.

**The rising epidemic of myopia will have far-reaching consequences for individuals and society unless we provide adequate treatment and care.**

**Figure:**
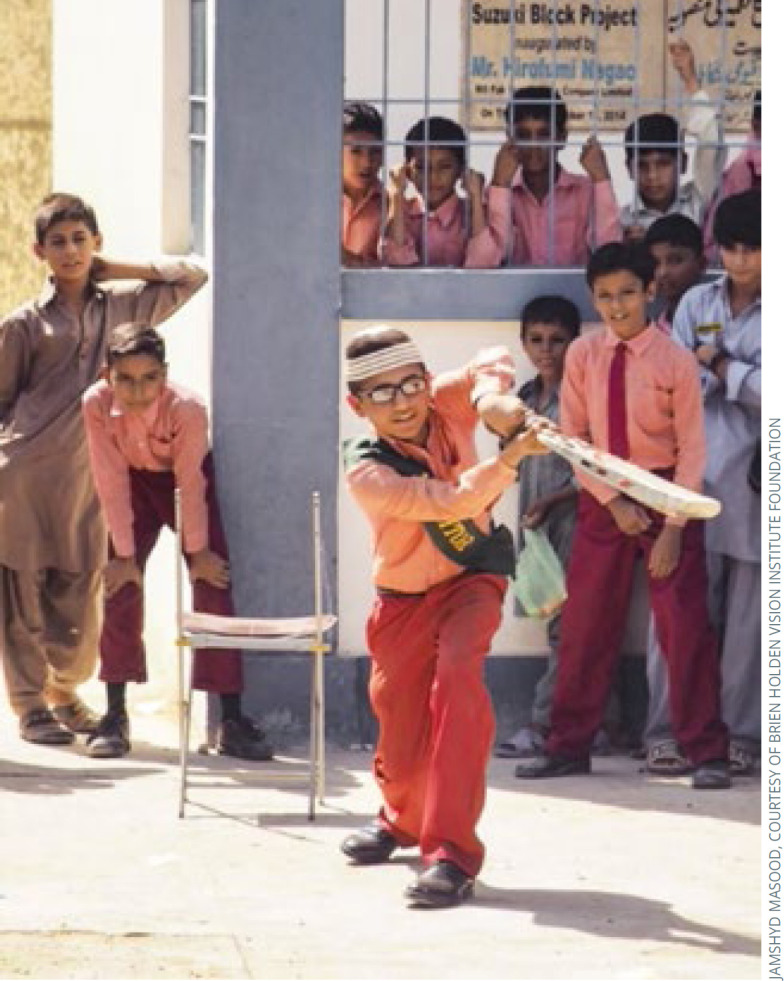
Spectacles improve academic performance and may assist in myopia control. PAKISTAN

Good distance vision is important for many activities; for example, recognising someone across the road, driving a vehicle and reading from a blackboard in school. The impact of uncorrected myopia varies depending on the age of the person affected. Those who develop high myopia, defined as ≤ −5 dioptres (D) are at increased risk of cataract, open-angle glaucoma, myopic macular degeneration and retinal detachment (p. 5), all of which has a personal and economic cost.

## The impact on children's education

Myopia usually starts to develop after the age of 10 years, but in East Asia it can start earlier. Uncorrected myopia can affect children's ability to learn in school and their quality of life.

An important benefit of providing school-aged children with spectacles is enhanced educational outcomes.

A randomised controlled trial among 20,000 children in 250 schools in Western China by Ma et al.^1^ reported that mathematics test scores at the end of a school year had improved significantly among the 1,153 children who had failed visual acuity screening and were offered free spectacles; the difference was the equivalent of half a semester of additional learning.

**“Myopia does not only affect educational outcomes; disadvantages arising from myopia also extend to quality of life and personal and psychological well-being.”**

An important finding in this study was that the beneficial effect of providing spectacles increased when there was a greater use of blackboards (rather than textbooks) for teaching in the classroom; this points to the impact of providing spectacles for myopia, as near-sighted children would be expected to benefit more from spectacles when trying to see a blackboard in the distance rather than a book that was close to them. Providing spectacles also exceeded the average educational impact observed in many other school-based health interventions in low- or middle-income countries, including dosing with iron to correct anaemia, giving vitamins and giving medications to eliminate parasites.^1^

These results are consistent with studies of varying designs, suggesting a causal association between better visual acuity and children's academic performance. In a prospective cohort study, Jan et al.^2^ have reported that Chinese children with better presenting vision at the beginning of the 7th grade had significantly better test scores at the end of the 9th grade (adjusted for baseline test performance). Additional studies are needed in school settings outside of China, and studies are also needed to determine whether provision of free spectacles can significantly improve other important indicators of children's academic success, such as remaining in school.

Although programmes of free spectacle delivery are shown to increase children's use of spectacles significantly,^1^,^3^ the impact has been limited by sub-optimal spectacle wearing, which may be 40% or lower.^1^,^4^ Effective methods to increase spectacle wear may vary from place to place, but are important to maximise the educational impact for children.

## The personal impact of myopia

Myopia does not only affect education outcomes; disadvantages arising from myopia also extend to quality of life and personal and psychological well-being and development, particularly when individuals develop high myopia.

Adolescents with vision impairment reported statistically significantly lower quality of life, psychosocial functioning and school functioning scores.^5^ Myopia has also been demonstrated to significantly increase levels of anxiety among adolescents,^6^ whereas studies in children have identified links between having myopia and experiencing low self-esteem. Dias et al.^7^ reported that children who experience more visual symptoms (e.g., tired eyes or headaches) tend to evaluate themselves less favourably in terms of their physical appearance, school work, social activities, and behavioural conduct. This finding is similar to another study in which more severe myopia was associated with lower self-esteem.^8^

Adolescents and younger children can experience social pressures against spectacle wear and may avoid wearing the spectacles they have been prescribed. These pressures have been identified in children across a range of contexts; for instance, children reported being teased or discriminated against (or being afraid of this) in studies from Brazil,^9^ India,^10^,^11^ Tanzania^12^ and Timor-Leste.^13^ Parents are also sensitive to social pressures and hesitate to obtain spectacles for their children due to the stigma associated with this.^14^

Studies from high-income countries have shown that adults with high myopia reported psychological, cosmetic, practical, and financial factors specifically related to myopia that affected their quality of life.^15^

## The economic impact on society

In 2015, uncorrected myopia was estimated to have caused $244 billion of potential lost productivity worldwide. Macular degeneration due to myopia was associated with another $6 billion of potential productivity loss.^16^ The greatest absolute economic burden was experienced in Asia.

There are very few economic evaluations of myopia correction and no economic evaluation of myopia prevention. However, disability weights have been used to estimate potential productivity loss as a proportion of the gross domestic product (GDP per person).^16^ In 2012, Fricke et al. found that the global cost of establishing educational and refractive care facilities was a small fraction of the projected global lost productivity for all types of uncorrected refractive error.^17^ In Singapore, for example, the cost of providing myopia care for adults in 2009 was an average of US $709 per person,^18^ which is less than 2% of the GDP per person at the time. By comparison, the disability weights associated with blindness (0.187) and moderate visual impairment (0.031) due to uncorrected refractive error^19^ represent potential lost productivity of 18.7% and 3.1% of GDP per capita, respectively. Spending less than 2% of GDP per capita to avoid a bigger loss in productivity therefore makes economic sense.

## Conclusion

The potential improvements in academic achievement for children who receive myopia correction, along with associated quality of life and psychosocial functioning gains – together with the economic case for myopia treatment in terms of productivity – speak to the need for interventions. However, addressing parents' and children's concerns about wearing spectacles and making efforts to maximise spectacle wear are critical for realising the economic or academic benefits of correcting myopia.
